# Telerehabilitation with Web-Based Exercises for Individuals with Postural Problems: Digital Touch to Posture Disorders—A Randomized Controlled Study on Telerehabilitation for Postural Problems

**DOI:** 10.3390/healthcare14132008

**Published:** 2026-07-06

**Authors:** Duygu Korkem Yorulmaz, Alperen Yazıtaş, Mehmet Furkan Cantürk, Tezel Yıldırım Şahan

**Affiliations:** 1 Gulhane Faculty of Physiotherapy and Rehabilitation, University of Health Sciences, Ankara 06018, Türkiye; fzttezel@gmail.com; 2 Gulhane Institute of Health Sciences, University of Health Sciences, Ankara 06018, Türkiye; alperenyazitas@gmail.com (A.Y.); m.furkancanturk26@gmail.com (M.F.C.)

**Keywords:** exercise, kyphosis, lordosis, posture, telerehabilitation

## Abstract

**Background:** Postural problems such as head forward posture, thoracic hyperkyphosis, and lumbar hyperlordosis, when seen together, further complicate postural control, increasing the importance of comprehensive approaches in treatment. This study aims to examine the effect of 6 weeks of telerehabilitation with web-based exercises and compare the home-based exercises in individuals with postural problems. **Trial Design:** A Randomized Controlled Study. **Methods:** A total of 34 volunteers with postural deformity among young adults were randomly divided into telerehabilitation (*n* = 17) and control (*n* = 17) groups. Craniovertebral, thoracic kyphosis, and lumbar lordosis angles of all individuals were evaluated with a smartphone application (Clinometer+ Bubble), hamstring and pectoral muscle shortness with a goniometer, and trunk muscle endurance with endurance tests created by McGill and Sorenson. Whilst the tele-rehabilitation group was provided with a video-based exercise program, the control group was advised to follow the same exercise program at home. Exercises were performed 3 days a week for 6 weeks, as 1-h sessions. Participants in the telerehabilitation group were followed up with a synchronized video conference. **Results:** A significant difference was observed in the telerehabilitation group in muscle shortness and the endurance tests (*p* < 0.05). Only a significant difference in left (*p* = 0.03) and right (*p* = 0.04) muscle shortness was observed in the home exercise group. Significant differences were observed in Craniovertebral and lumbar lordosis angles between groups (*p* < 0.05), with the telerehabilitation group showing better outcomes. The kyphosis angle, muscle shortness, and endurance test results between groups were found to be similar (*p* > 0.05). **Conclusions:** Six weeks of telerehabilitation can improve muscle shortness and trunk endurance in young adults with postural deformities. Both the exercise program using telerehabilitation and the home exercise program were beneficial for individuals with postural problems, with more favorable effects observed in the telerehabilitation group.

## 1. Introduction

Telerehabilitation is a type of rehabilitation in which information and communication technology allows for the remote provision of physical rehabilitation services, including assessment, monitoring, intervention, supervision, training, counseling, etc. [1]. Telerehabilitation delivers information and communication technologies, including videoconferencing, web-based platforms, and mobile applications. It facilitates the provision of assessment, monitoring, education, supervision, and therapeutic interventions without requiring face-to-face contact between patients and healthcare professionals [1,2]. Recent systematic reviews and meta-analysis trials have demonstrated that telerehabilitation can produce clinically meaningful improvements in pain, physical function, exercise adherence, and health-related quality of life, with outcomes comparable to those of conventional face-to-face rehabilitation in selected populations [1,2]. Furthermore, evidence suggests that remotely delivered exercise interventions may positively influence sensorimotor and postural functions. For example, Suner et al. reported significant improvements in trunk proprioception and core muscle endurance following an online exercise program, supporting the use of telerehabilitation as a promising approach for the management of posture-related musculoskeletal problems [3]. Given the high prevalence of postural disorders and their association with pain, functional limitations, reduced physical activity, and diminished quality of life, effective and accessible rehabilitation strategies are required.

Thoracic kyphosis refers to the physiological posterior convex curvature of the thoracic spine and is generally considered normal when ranging between 20° and 40° in healthy adults, although age-related increases have been reported across the lifespan [4]. Deviations from these normative values may contribute to postural impairments, altered spinal loading, reduced physical performance, and musculoskeletal symptoms, highlighting the importance of early assessment and intervention [4]. Increased thoracic kyphosis can significantly alter the position of the scapula and tends to increase scapular protraction and anterior tilt, decreasing range of motion and function of the shoulder [4,5]. Lumbar lordosis refers to the inward (ventral) curvature of the lumbar spine [6]. One of the most crucial components of the spine is lumbar lordosis. Because of its unusual location and direct contact with the pelvis, it is very significant. Strength against the compressive forces of gravity is provided by lumbar lordosis [7]. It has been suggested that postural pain, radiculopathy, and facet pain are mostly caused by increased lordosis [8]. Pacheco et al. discovered that among college students, 97% had pelvic asymmetry, 85.7% had cervical hyperlordosis, 74.2% had thoracic kyphosis, and 65.7% had lumbar hyperlordosis [9]. Ardakani et al. reported that the female-to-male ratio of thoracic hyperkyphosis was approximately 2:1 and that this postural abnormality was present in 38% of individuals aged 20–50 years and 35% of those aged 20–64 years [10]. Given its relatively high prevalence, thoracic hyperkyphosis represents an important public health concern. Excessive thoracic kyphosis has been associated with impaired postural alignment, altered spinal biomechanics, reduced physical performance, musculoskeletal pain, and diminished quality of life. Therefore, effective rehabilitation strategies aimed at improving posture, flexibility, and trunk muscle function are essential for preventing long-term functional limitations. Conventional face-to-face rehabilitation programs for postural disorders typically require supervised sessions conducted two to three times per week over a period of 6–8 weeks or longer. Although these interventions can be effective, barriers such as travel time, transportation costs, scheduling difficulties, and limited access to rehabilitation services may reduce adherence and restrict treatment accessibility for many individuals [10].

In recent years, the increasing integration of digital health technologies into rehabilitation practice has expanded access to therapeutic interventions for individuals with musculoskeletal disorders. Among these approaches, telerehabilitation has emerged as a promising method for delivering assessment, monitoring, education, and exercise-based interventions remotely. With the advantage of providing rehabilitation services regardless of geographical location, telerehabilitation appears to be an effective and accessible treatment option for individuals with musculoskeletal conditions [2]. According to Khruakhorn et al., telerehabilitation with simultaneous message feedback and visual demonstration enhanced kyphosis angle, physical function, and quality of life [11]. Evidence supporting the effectiveness of telerehabilitation in musculoskeletal disorders has continued to grow. In a recent meta-analysis, Huang et al. [12] reported that telerehabilitation was more effective than home-based exercise programs in improving range of motion, functional outcomes, and quality of life, particularly among individuals with scapular and shoulder disorders. Likewise, Mbada et al. demonstrated significant improvements in general health status and quality of life among individuals with chronic low back pain following telerehabilitation interventions. Although these findings support the use of telerehabilitation in various musculoskeletal conditions, evidence regarding its effectiveness in individuals with postural disorders remains limited, highlighting the need for further investigation in this population [13].

Previous studies have demonstrated that telerehabilitation can improve posture, spinal alignment, flexibility, muscle endurance, physical function, and quality of life in individuals with specific postural impairments, including forward head posture and thoracic hyperkyphosis [12,14]. However, most previous studies have focused on a single postural deviation rather than the combination of multiple postural disorders that commonly coexist in clinical practice. Furthermore, there are very few randomized controlled trials directly comparing telerehabilitation with home-based exercise programs in this population; this makes it difficult to determine the added value of remote monitoring and feedback. Furthermore, significant heterogeneity in study design, participant characteristics, outcome measures, and intervention delivery methods has led to inconsistent findings and limited the generalizability of the current evidence. Consequently, the current literature does not provide sufficient evidence regarding the comprehensive management of postural disorders via telerehabilitation, and well-designed additional randomized controlled trials have been recommended [12,14]. Previous exercise-based telerehabilitation studies have reported measurable improvements in posture, flexibility, muscular endurance, and functional outcomes following interventions lasting 6–8 weeks, suggesting that this duration may be sufficient to induce clinically meaningful changes [11,12,14]. In addition, telerehabilitation offers several practical advantages, including improved access to rehabilitation services, reduced travel burden, and greater scheduling flexibility [15]. Therefore, this randomized controlled trial aimed to compare the effects of a six-week internet-based telerehabilitation program with a home-based exercise program on postural alignment, muscle shortening, and trunk muscle endurance in individuals with postural disorders.

## 2. Materials and Method

### 2.1. Participants

This randomized controlled study included 34 young adults aged 18–45 years who presented with postural disorders and associated spinal pain symptoms. Participants were recruited from individuals who applied to the Spinal Health Unit of the Faculty of Physical Therapy, University of Health Sciences, between June and October 2025. The clinical presentation of the participants included forward head posture, thoracic hyperkyphosis, lumbar hyperlordosis, or combinations of these postural deviations, accompanied by neck, thoracic, or low back pain.

**Figure 1:**
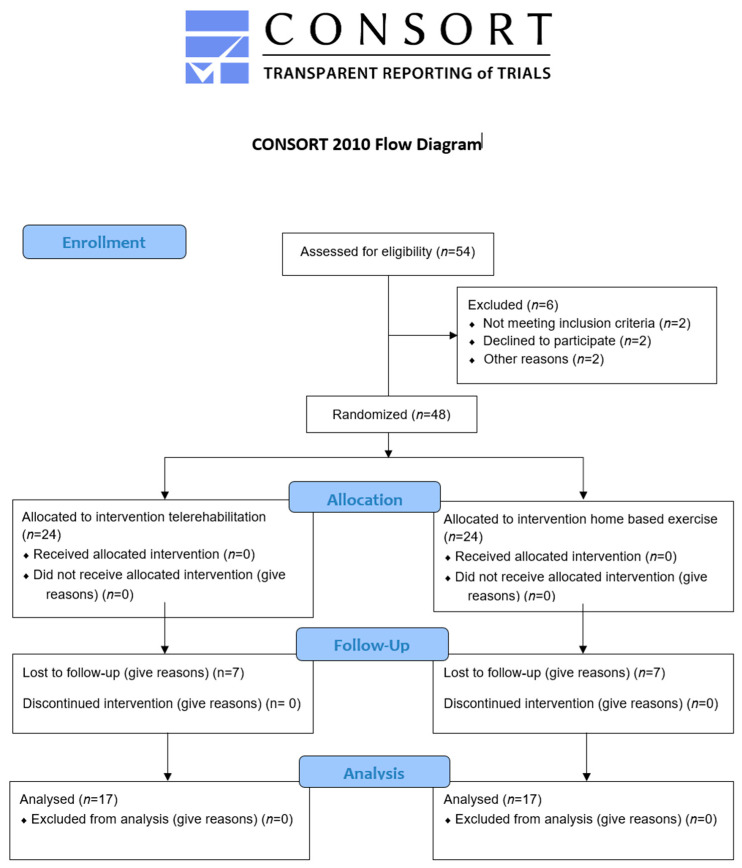
Flow Diagram.

Participants were allocated to the intervention or control group in a 1:1 ratio using a computer-generated simple randomization sequence (Random.org). An independent researcher generated and concealed the allocation sequence using sequentially numbered, opaque, sealed envelopes. Participants were recruited voluntarily, and a written informed consent form was signed. Helsinki principles were followed, and ethical approval was obtained from the University of Health Sciences, Non-Interventional Clinical Research Ethics Committee with the decision number 2025–228. The clinical trial number for the study was obtained on 25 May 2025 with the number NCT06964750 (https://clinicaltrials.gov/expert-search?term=NCT06964750, accessed on 19 March 2025). Participants were informed that they could withdraw from the study at any time without providing a reason and without any consequences regarding future healthcare services. Following postural assessment, eligible participants with postural deformities were randomly assigned to the study groups. The participant groups were divided into two groups: telerehabilitation (*n*: 17), and home-based group (*n*: 17) (Figure 1). To minimize potential sources of bias, outcome assessments were conducted by an independent physiotherapist who was not involved in treatment delivery, participant recruitment, or group allocation procedures. Participants were instructed not to disclose their group allocation during the assessment sessions.

An a priori power analysis was conducted using G*Power software (version 3.1.9.4) to determine the required sample size based on the primary outcome data from Cho et al. (2015) [16]. For the calculation, the means and standard deviations of the two groups yielded a Cohen’s d effect size of 0.90. With a significance level (alpha) of 0.05, a power (1 – β) of 0.80, and an allocation ratio of 1:1 for a one-tailed Wilcoxon-Mann–Whitney test, the minimum required sample size was determined to be 34 participants (17 per group), achieving an actual power of 0.81.

Participants were eligible for inclusion if they voluntarily agreed to participate in the study and had a body mass index (BMI) below 30 kg/m^2^. Additional inclusion criteria required the presence of cervical, thoracic, or lumbar pain greater than 3 on the Visual Analog Scale (VAS), pain symptoms had persisted for at least six months, and a craniovertebral angle measured as less than 52 degrees, indicating forward head posture. Individuals were excluded if they presented with acute or chronic neck, back, or low back pain of a severity that would interfere with testing procedures, or if they were regular exercisers or amateur/professional athletes, as habitual training could confound postural and endurance outcomes. Participants were also excluded if they had a known spinal deformity such as scoliosis, a history of spinal surgery, or any neurological, orthopedic, or congenital disorder affecting the spine. Additional exclusion criteria included visual or hearing impairments that could limit the ability to follow instructions or perform assessments, as well as having received physiotherapy treatment within the previous six months [11,12,14].

All assessments and treatments were carried out by physiotherapists with at least 10 years’ experience in the field of spinal health.

### 2.2. Assessment Parameters

The primary outcomes were craniovertebral angle, thoracic kyphosis angle, and lumbar lordosis angle, representing the main indicators of postural alignment. Secondary outcomes included hamstring muscle shortness, pectoralis muscle shortness, lateral trunk endurance, trunk extensor endurance (Sorensen test), and trunk flexor endurance (McGill endurance test). All outcomes were assessed at baseline and immediately after the 6-week intervention. The primary analysis metric was the change from baseline to post-intervention.

#### 2.2.1. Spine Angles

Individuals with postural issues will have their craniovertebral, thoracic kyphosis, and lumbar lordosis angles, hamstring and pectoral muscle shortening, and trunk endurance assessed. Two reference points were used to determine the craniovertebral angle: a line that runs from the ear’s tip to the seventh cervical vertebra (C7) and another horizontal line parallel to the ground that only goes through the spinous apophysis of C7 [17,18,19]. Craniovertebral, thoracic kyphosis, and lumbar lordosis angles: The “Clinometer + bubble level” application (Version 4.9.4, plaincode™, Stephanskirchen, Germany), which is a smartphone application available on the Google Play Store and validated by Elpeze et al., was used [17,18,19].

#### 2.2.2. Shortness Tests

Hamstring Muscle Shortness Test: The individual was asked to perform active knee extension while positioned supine with the hip joint in 90 degrees of flexion. The lower back and non-measured knee were fixed on the bed. The individual was asked to slowly extend the hip and knee. When there is no knee tremor, the value measured with the goniometer was recorded in degrees. Hamstring muscle shortness was determined in individuals with active knee extension of less than 70 degrees [20].

Pectoral Muscle Shortness Test: The individual was supine with knees slightly flexed. In this position, the patient was allowed to contact the bed freely with their hands behind their back, maintaining the smoothness of the lumbar vertebrae, and without forcing the elbows. If the elbows are not in contact with the bed, the distance between the lateral epicondyle of the humerus and the bed was measured with a tape measure [21].

#### 2.2.3. Trunk Endurance Tests

The Lateral Bridge Test, Sorensen Test, and Trunk Flexors Endurance Test (Mc-Gill Endurance Test) were used. Measurements were recorded in seconds using a chronometer. The tests were planned to be terminated when the test position was disturbed. Each measurement was performed 2 times, and the best measurement was used for statistical analysis.

Lateral Bridge Test: The lateral trunk muscles’ static endurance was assessed using the lateral bridge test. The test required the individuals to elevate their bodies on their forearms and toes, turn sideways to their right, and stay in this position. On the opposite side, it was repeated [22].

Sorensen Test: This test was used to assess the static endurance of the trunk extensors. The subjects were positioned face down, with their pelvis, hips, and knees resting on the bed. They were instructed to extend their upper bodies straight forward over the edge of the table [23].

Flexors Endurance Test (Mc-Gill Endurance Test): Subjects were asked to assume a supine position with knees bent at 90°. They will then be asked to raise their head and shoulders until the lower angles of the scapula cross the table. The person was allowed to correct this position once. The number of seconds this position is maintained was measured by the observer using a hand-held stopwatch [24].

#### 2.2.4. Telerehabilitation Exercise Program

Participants allocated to the telerehabilitation group received a structured exercise program designed using MuscleWiki (https://musclewiki.com; accessed on 20 March 2026). The program targeted major upper-body and postural muscle groups and was standardized across participants. Exercise sessions were performed 3 days per week for 6 consecutive weeks under real-time supervision. Sessions were delivered synchronously via WhatsApp video group calls, led by a physiotherapist. During each session, participants were monitored for correct exercise performance, posture, compensatory movements, breathing control, and adherence to prescribed dosage. Real-time verbal feedback and corrections were provided when necessary. Each session lasted approximately 45 min, including warm-up, main exercise program, rest intervals, and cool-down. During the first 2 weeks, exercises were performed as 2 sets × 10 repetitions. From week 3 onwards, exercise volume was progressed to 3 sets × 15 repetitions. Progression was individualized based on participant tolerance, symptom response, fatigue level, and movement quality [25] (Table 1).

#### 2.2.5. Home-Based Exercises

**Figure 2:**
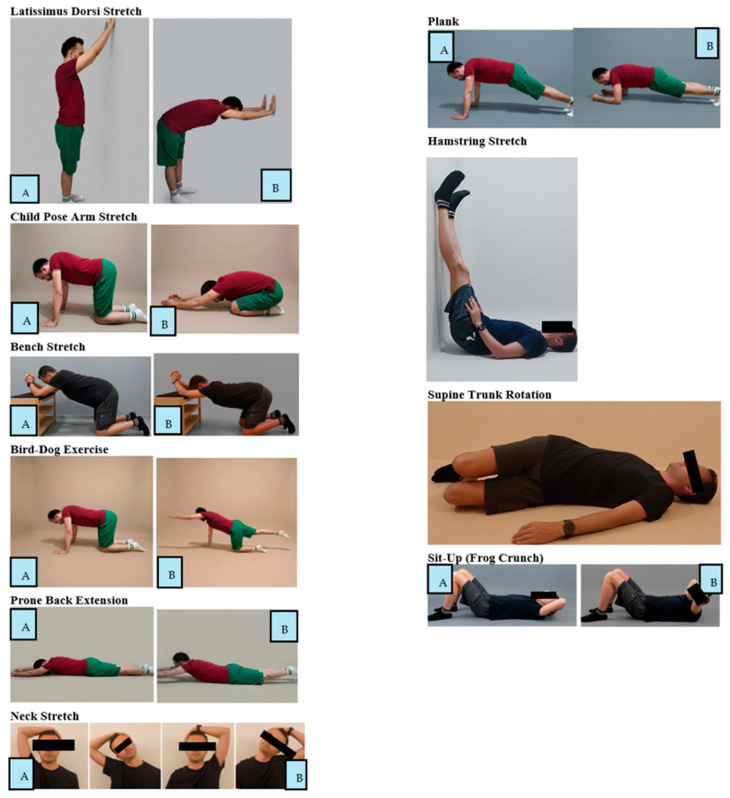
Exercise Brochure. (**A**); Start position, (**B**); End position; The detailed descriptions of the exercises and their demonstration links are provided in Table 1.

Participants in the home-based exercise group performed the same exercise program independently at home. To ensure consistency between groups, the exercise content, duration, and progression schedule were identical to the telerehabilitation intervention. Each participant received a written and illustrated exercise brochure including step-by-step instructions, starting position, movement execution, breathing pattern, number of repetitions, and precautions for each exercise (Figure 2). Participants were instructed to perform the program 3 days per week for 6 weeks. Session duration was approximately 45 min. During weeks 1–2, exercises were completed as 2 sets of 10 repetitions. Beginning in week 3, the program was progressed to 3 sets of 15 repetitions. Adherence was monitored using an online Excel-based exercise diary, where participants recorded whether each session had been completed. Weekly follow-up messages were sent by the physiotherapist to encourage compliance and answer questions related to exercise performance [12,26,27].

#### 2.2.6. Adverse Event Monitoring

Potential adverse events were defined as any undesirable symptom occurring during or after the exercise sessions, including increased neck, thoracic, or low back pain, dizziness, excessive fatigue, muscle strain, shortness of breath, or any symptom requiring modification or discontinuation of the exercise program. All adverse events reported throughout the intervention period were documented and reviewed by the research team.

### 2.3. Statistical Analysis

The IBM SPSS Statistics package, version 23.0 (SPSS Inc., Chicago, IL, USA), was used for data management and analysis. For quantitative data, the mean and standard deviation (SD) are presented. For qualitative variables, percentages (%) are displayed. *p* < 0.05 was the threshold for statistical significance. Using the “One-Sample Kolmogorov–Smirnov Test” and creating a “Histogram” in the statistical analysis test choice, the suitability of all the data for a normal distribution was assessed. Within-group differences were compared using non-parametric testing using the “Wilcoxon Signed-Rank Test.” To demonstrate group differences, the “Mann–Whitney U-Test” was employed. Additionally, “Pearson’s Chi-Square” analysis was used to look at variations between categorical variables, and *p* < 0.05 was chosen as the threshold for statistical significance.

The CONSORT reporting checklist is available in the Supplementary Materials [28,29].

## 3. Results

When the telerehabilitation and home-based exercise groups were compared before the exercises, there was no difference between the two groups in terms of demographic characteristics (*p* > 0.05) (Table 2). Among the demographic characteristics, only age was found to be different (*p* = 0.016).

In terms of craniovertebral, thoracic kyphosis, lumbar lordosis angles, muscle shortness, and endurance tests for pre-interventions, there was no difference between the telerehabilitation and home-based exercise groups before the exercises (*p* > 0.05). There was a difference only in the measurement of right pectoral muscle shortness (*p* = 0.03) (Table 3).

When comparing within groups, a significant difference was observed in the telerehabilitation group in terms of muscle shortening and in the lateral bridge left side test of the endurance tests after exercises (*p* < 0.05). Only a significant difference in left (*p* = 0.03) and right (*p* = 0.04) hamstring muscle shortening was observed after 6 weeks in the home exercise group (Table 4). When comparing the results of the 6-week exercise programs between the telerehabilitation and home exercise groups, significant differences were observed in CVA and LA angles (*p* < 0.05), with the telerehabilitation group showing better outcomes. When examined in terms of KA, muscle shortness, and endurance tests, the results between the groups were found to be similar (*p* > 0.05) (Table 5).

## 4. Discussion

The purpose of this study was to compare the effects of home-based workouts on postural deformities in people with posture issues and to investigate the 6-week impact of telerehabilitation with web-based activities. After the six-week intervention, the telerehabilitation group showed significant improvements in trunk muscle endurance and reductions in muscle shortening. Similarly, participants in the home exercise group also showed a significant reduction in hamstring muscle shortening following the intervention. When the telerehabilitation and home exercise groups were compared after treatment, telerehabilitation was found to be more effective on spinal angles.

### 4.1. Angles

Clinical guidelines suggested that multidisciplinary non-surgical management for most musculoskeletal spinal conditions [30]. Telerehabilitation-based exercise approaches are valid and reliable tools among non-surgical methods used in adults with spinal pain [31,32]. Özel et al. demonstrated in their studies that structured exercise therapy, when applied with or without supervision in rehabilitation, can improve outcomes for chronic nonspecific neck pain [32]. Khruakhorn et al. showed that an 8-week telerehabilitation program was effective and efficient in improving thoracic kyphosis and craniovertebral angle in elderly individuals with a thoracic kyphosis angle greater than 45 degrees and forward head posture, showing no difference in effectiveness and efficiency compared to in-person treatment at the clinic [11]. The heterogeneous response across postural parameters observed in the present study is consistent with previous research suggesting that spinal alignment adaptations occur at different rates depending on the targeted anatomical region and underlying biomechanical impairments. In particular, changes in craniovertebral angle and lumbar lordosis angle appeared to be more responsive to the intervention than other postural parameters, suggesting that certain aspects of posture may be more amenable to short-term exercise-based rehabilitation, whereas thoracic kyphosis may require longer intervention periods to achieve clinically meaningful adaptations [11,30,32]. Furthermore, this study is one of the few studies that examines all spinal angles together with 6 weeks of telerehabilitation-based exercises, and it is thought that it will guide future studies in this direction.

### 4.2. Hamstring Muscle Shortness

Improvements in hamstring flexibility were observed following both intervention approaches, suggesting that hamstring extensibility may be particularly responsive to structured stretching programs regardless of the mode of delivery. Previous studies have reported that regular hamstring stretching can improve flexibility and lumbopelvic mobility through adaptations in stretch tolerance and neuromuscular function [33,34,35]. The lack of a between-group difference in the present study may indicate that, unlike more technique-dependent exercises, hamstring stretching can be performed effectively after appropriate instruction without requiring continuous supervision. This finding is clinically relevant because it suggests that simple flexibility interventions targeting the posterior kinetic chain may be successfully implemented through both supervised and home-based rehabilitation models. Nevertheless, longer intervention periods and larger samples may be required to determine whether remote supervision provides additional benefits for flexibility-related outcomes [36].

### 4.3. Pectoral Muscle Shortness

The findings related to pectoral muscle length suggest that upper-quarter flexibility may be influenced by factors beyond exercise prescription alone. Stretching interventions targeting the pectoral musculature often require appropriate scapular positioning and control of compensatory trunk movements to achieve the desired tissue loading [37,38]. Consequently, interventions that incorporate exercise instruction and movement monitoring may facilitate the correct execution of stretching exercises. However, the present findings should be interpreted with caution, as the observed changes were not accompanied by clear evidence of the superiority of one intervention approach over the other. It is possible that the relatively short intervention duration and limited sample size reduced the ability to detect potential differences between groups. Future studies with larger samples and longer follow-up periods are warranted to further investigate the influence of different rehabilitation delivery models on upper-quarter flexibility outcomes.

### 4.4. Trunk Endurance Tests

The literature indicates that meaningful improvements in trunk endurance occur when exercise programs last longer than six weeks and include structured, progressive loading [39,40,41]. Hoppes et al. demonstrated that a core stabilization program significantly improved trunk extensor and flexor endurance in adults with low back pain [39]. Geçit et al. similarly emphasized that an eight-week progressive core intervention resulted in significant improvements in trunk flexor and lateral endurance [41]. A 2024 randomized trial evaluating the effects of structured core training showed significant improvements across Sorensen, lateral bridge, and trunk flexor endurance tests after a nine-week protocol, underscoring that endurance gains are strongly time- and dose-dependent [42]. Although the intervention period was relatively short, the findings suggest that trunk endurance outcomes may require longer or more progressively loaded exercise programs to demonstrate consistent changes across all endurance parameters. The limited improvement observed in lateral trunk endurance may reflect an early adaptation to postural and core stabilization exercises; however, this interpretation should be made cautiously because similar changes were not observed across all trunk endurance tests. Future studies should consider longer intervention periods, progressive loading strategies, and follow-up assessments to determine whether telerehabilitation can produce sustained and clinically meaningful improvements in trunk muscle endurance.

### 4.5. Telerehabilitation- Home-Based Exercises

The findings observed in the telerehabilitation group may be related to the additional benefits of real-time supervision, exercise adherence monitoring, immediate feedback, and correction of compensatory movements during exercise performance. These factors may contribute to improved exercise quality and adherence, which are considered important components of successful postural rehabilitation. Accordingly, emerging evidence suggests that remotely supervised exercise programs may offer advantages over unsupervised home-based interventions for outcomes requiring precise movement control and postural correction [43,44,45]. Recent studies in individuals with postural neck pain and forward head posture have similarly demonstrated that telerehabilitation produces superior gains in craniovertebral angle and deep cervical flexor function compared with unsupervised or conventional programs, underscoring the importance of guided motor-learning strategies for postural correction [2,32,45].

However, parameters such as thoracic kyphosis, overall body strength, and overall muscle shortening generally respond more slowly for 6 weeks. Therefore, the absence of differences between groups in terms of kyphosis angle and overall strength after 6 weeks of telerehabilitation and home exercise programs indicates that longer-term application is necessary. The superior outcomes observed for craniovertebral angle and lumbar lordosis angle in the telerehabilitation group suggest that postural parameters requiring precise movement control and continuous exercise supervision may particularly benefit from remotely supervised rehabilitation. These findings are consistent with previous studies reporting that structured telerehabilitation programs can facilitate improvements in postural alignment through enhanced exercise adherence, movement quality, and real-time feedback during exercise performance [2,11,45].

Furthermore, this study is one of the few studies that examines all spinal angles together with 6 weeks of telerehabilitation-based exercises, and it is thought that it will guide future studies in this direction. This study has several limitations that should be considered when interpreting the findings. First, therefore, future randomized controlled trials with larger samples are warranted to further investigate the effectiveness of telerehabilitation in individuals with postural disorders. Second, the intervention duration was limited to six weeks, which may be insufficient to elicit substantial changes in global trunk endurance or spinal alignment parameters that typically respond over longer timeframes. Third, the sample consisted of patients from a single center, which may limit the generalizability of the results to broader populations with different physical activity levels, occupational demands, or postural habits. Fourth, adherence to the home-based exercise program was monitored through self-report, which is subject to reporting bias. Fifth, although both groups performed the same exercise programme, participants in the telerehabilitation group received real-time supervision and feedback from the physiotherapist. Therefore, motivational factors and increased engagement associated with synchronous interaction may have contributed to exercise adherence and treatment outcomes. Future studies should consider assessing motivation and adherence levels to better determine their potential influence on intervention effectiveness. Finally, as this trial focused on short-term outcomes, the long-term sustainability of postural improvements remains unknown. Future research should incorporate follow-up assessments to evaluate retention, relapse, or further adaptation over time.

## 5. Conclusions

Both telerehabilitation and home-based exercise programs were associated with improvements in several postural and functional outcomes in individuals with postural disorders. However, telerehabilitation demonstrated greater improvements in craniovertebral angle and lumbar lordosis angle, suggesting that remotely supervised rehabilitation may provide additional benefits for selected aspects of postural alignment. No significant between-group differences were observed for thoracic kyphosis, muscle shortness, or trunk endurance outcomes. These findings support the use of telerehabilitation as a feasible alternative to home-based exercise for the management of postural disorders.

In future studies, extending the intervention period, incorporating gradual loading, and objective compliance monitoring could further enhance the benefits observed in this study and contribute to more comprehensive postural and neuromuscular improvements.

## Figures and Tables

**Table 1:** Telerehabilitation Exercise Protocol.

Exercise	Target Muscle Group	Procedure	Frequency	Exercise Dose (Sets × Repetitions)	Hold Time	Rest Interval	Progression
Latissimus Dorsi Stretch	Latissimus dorsi, shoulder complex	Standing facing a wall, both hands placed flat against the wall; the body is lowered slowly until a stretch is felt. https://musclewiki.com/tr-tr/exercise/lats-stretch-variation-one (accessed on 20 March 2026)	3 days/week	2 × 10 repetitions	10 s	20 s	Hold duration increased to 15–20 s as tolerated.
Child Pose Arm Stretch	Latissimus dorsi, thoracic extensors	From quadruped position, sit back onto heels while extending arms forward and lowering forehead to the floor. https://musclewiki.com/tr-tr/exercise/child-pose-arms-extended-left-right/ (accessed on 20 March 2026)	3 days/week	2 × 10 repetitions	10 s	20 s	Hold duration increased progressively.
Bench Stretch	Latissimus dorsi, shoulder external rotators	Kneeling in front of a bench with elbows supported; trunk lowered while maintaining shoulder external rotation. https://musclewiki.com/tr-tr/exercise/lat-and-shoulder-external-rotation-stretch-1-kneeling-dowel (accessed on 20 March 2026)	3 days/week	2 × 10 repetitions	10 s	20 s	Range of motion gradually increased.
Bird-Dog Exercise	Core stabilizers, multifidus, and gluteal muscles	From quadruped position, extend the contralateral arm and leg while maintaining trunk stability. https://musclewiki.com/tr-tr/exercise/core-stability-1-crosslateral-limb-raise-4pt-position (accessed on 20 March 2026)	3 days/week	2 × 10 repetitions/side	5–10 s	30 s	Hold time increased from week 3.
Prone Back Extension	Thoracic and lumbar extensors, gluteal muscles	In the prone position, lift chest and arms from the floor while activating gluteal muscles. https://musclewiki.com/tr-tr/exercise/swipe-around-prone/ (accessed on 20 March 2026)	3 days/week	2–3 × 10^–15^ repetitions	–	30 s	Repetition volume increased progressively.
Neck Stretch	Cervical muscles, upper trapezius	Standing upright, gently pull the head toward the shoulder using hand assistance. https://musclewiki.com/tr-tr/exercise/traps-stretch-variation-one (accessed on 20 March 2026)	3 days/week	2 × 10 repetitions/side	10 s	20 s	Hold duration increased according to tolerance.
Plank	Core stabilizers, abdominal muscles	Forearm plank, maintaining neutral spinal alignment. https://musclewiki.com/tr-tr/workouts/beginner-bodyweight-workout (accessed on 20 March 2026)	3 days/week	3 sets	20–60 s (maximum tolerated duration)	30 s	Hold duration increased weekly.
Hamstring Stretch	Hamstring muscle group	Supine position with one leg elevated against a wall while maintaining knee extension. https://musclewiki.com/tr-tr/exercises/hamstrings/stretches/ (accessed on 20 March 2026)	3 days/week	2 × 10 repetitions/side	10 s	20 s	Hold duration increased progressively.
Supine Trunk Rotation	Lumbar spine, trunk rotators	Supine position with knees flexed; pelvis and knees rotated alternately to each side. https://musclewiki.com/tr-tr/exercise/lower-back-stretch-variation-three (accessed on 20 March 2026)	3 days/week	2 × 10 repetitions/side	5 s end-range	20 s	Range of motion gradually increased.
Sit-Up (Frog Crunch)	Abdominal muscles	Supine position with knees flexed; trunk flexion performed through abdominal muscle contraction. https://musclewiki.com/tr-tr/exercise/frog-crunch (accessed on 20 March 2026)	3 days/week	2–3 × 10^–15^ repetitions	–	30 s	Sets and repetitions increased from week 3.

Note. All exercises were performed three times per week for six weeks. Stretching exercises were conducted within a pain-free range of motion. Progression was individualized according to participant tolerance and performance. Participants were instructed to maintain normal breathing during all exercises and discontinue any exercise that provoked pain or discomfort.

**Table 2:** Comparison of demographic characteristics between the groups.

Variables	Telerehabilitation Group (*n* = 17) (Mean ± SD)	Home-Based Exercise Group (*n* = 17) (Mean ± SD)	*p*
Age(year)	22.17 ± 1.28	23.94 ± 2.04	**0.016 ^a^**
Height (cm)	167.11 ± 8.95	169.23 ± 8.67	0.413 ^a^
Weight (kg)	62.23 ± 8.85	68.00 ± 12.85	0.170 ^a^
BMI (kg/m^2^)	22.26 ± 2.57	23.74 ± 4.32	0.357 ^a^
**Gender *n* (%)**			0.654 ^b^
Man	3 (17.6)	5 (29.4)	
Woman	14 (82.4)	12 (70.6)	
**Pain *n* (%)**			0.098 ^b^
None	1 (5.9)	7 (41.2)	
Low back	12 (70.6)	8 (47.1)	
Neck	3 (17.6)	1 (5.9)	
Upper back	1 (5.9)	1 (5.9)	
Physical Activity n (%)			0.300 ^b^
Yes	6 (35.3)	9 (52.9)	
No	11 (64.7)	8 (47.1)	

BMI: Body Mass Index; ^a^: Mann–Whitney U-Test; ^b^: Chi-Square Test; n = participants; % percentage.

**Table 3:** Pre-intervention intra-group comparison of craniovertebral angle, kyphosis angle, lordosis angle, muscle shortness, and endurance test results.

Variables	Telerehabilitation Group (*n*= 17) (Mean ± SD)	Home-Based Exercise Group (*n*= 17) (Mean ± SD)	Z	*p*
**Angles (Degree)**				
CVA	29.47 ± 9.12	34.58 ± 7.65	−1.51	0.120
KA	19.29 ± 4.39	21.68 ± 6.07	−1.36	0.170
LA	22.52 ± 5.94	17.76 ± 8.27	−1.74	0.080
**Shortness (centimeter)**				
Hamstring(L)	29.58 ± 10.08	30.76 ± 15.29	−0.34	0.720
Hamstring (R)	30.41 ± 11.74	34.17 ± 15.73	−0.57	0.560
Pectoral (L)	2.11 ± 1.40	0.79 ± 1.33	−2.61	0.090
Pectoral (R)	2.17 ± 1.38	0.76 ± 1.48	−2.99	0.030 *
**Endurance (second)**				
Lateral Bridge (L)	26.73 ± 14.90	36.65 ± 23.74	−1.01	0.310
Lateral Bridge (R)	36.40 ± 21.04	34.41 ± 24.19	−0.49	0.610
Sorensen	27.22 ± 14.47	42.74 ± 29.84	−1.87	0.060
Mc-Gill	42.13 ± 24.28	51.08 ± 26.06	−0.98	0.320

CVA: Craniovertebral Angle; KA: Kyphosis Angle; LA: Lordosis Angle; SD: Standard Deviation; Z: inter-group comparison; *p*: Mann–Whitney U Test; *p* * < 0.05.

**Table 4:** Intragroup comparison of craniovertebral angle, kyphosis angle, lordosis angle, muscle shortness, and endurance tests.

Variables	Telerehabilitation Group (*n* = 17) (Mean ± SD)		Z	*p*	Home-Based Exercise Group (*n* = 17) (Mean ± SD)		Z	*p*
	Pre-test	Post-test			Pre-test	Post-test		
**Angles (Degree)**								
CVA	29.47 ± 9.12	28.44 ± 7.22	−0.220	0.82	34.58 ± 7.65	34.35 ± 4.87	−0.826	0.40
KA	19.29 ± 4.39	17.94 ± 4.09	−1.038	0.29	21.68 ± 6.07	21.14 ± 6.49	−0.802	0.42
LA	22.52 ± 5.94	21.41± 5.46	−0.483	0.62	17.76 ± 8.27	15.94 ± 6.87	−1.204	0.22
**Muscle Shortness**								
Hamstring (L) degree	29.58 ± 10.08	19.11 ± 10.49	−3.198	<0.001 *	30.76 ± 15.29	23.35 ± 12.36	−2.094	0.03 *
Hamstring (R) degree	30.41 ± 11.74	21.88± 13.16	−2.212	0.020 *	34.17 ± 15.73	26.88 ± 12.35	−2.046	0.04 *
Pectoral (L) **centimeter**	2.11 ± 1.40	0.79± 0.91	−3.133	**0.002** *	0.79 ± 1.33	0.70 ± 1.12	−0.756	0.45
Pectoral (R) **centimeter**	2.17 ± 1.38	0.82 ± 10.02	−3.074	**0.002** *	0.76 ± 1.48	0.52 ± 0.94	−1.134	0.25
**Endurance (second)**								
Lateral Bridge (L)	26.73 ± 14.90	35.54 ± 19.05	−2.783	0.005 *	36.65 ± 23.74	38.02 ± 23.096	−664	0.50
Lateral Bridge (R)	36.40 ± 21.04	36.47 ± 20.32	−0.341	0.733	34.41 ± 24.19	31.08 ± 13.06	−0.317	0.75
Sorensen	27.22 ± 14.47	38.46 ± 21.68	−1.931	0.053 *	42.74 ± 29.84	41.25 ± 27.43	−0.035	0.97
Mc-Gill	42.13 ± 24.28	45.21 ± 29.54	−0.284	0.776	51.08 ± 26.06	49.99 ± 6.93	−0.105	0.91

CVA: Craniovertebral Angle; KA: Kyphosis Angle; LA: Lordosis Angle; SD: Standard Deviation; Z: intra-group comparison; *p*: Wilcoxon Test; *p* * < 0.05.

**Table 5:** Post-intervention, inter-group comparison of craniovertebral angle, kyphosis angle, lordosis angle, muscle shortness, and endurance tests.

Variables	Telerehabilitation Group (*n* = 17) (Mean ± SD)	Home-Based Exercise Group (*n* = 17) (Mean ± SD)	Z	*p*
	Post-test	Post-test		
**Angles (Degree)**				
CVA	28.44 ± 7.22	34.35 ± 4.87	−2.041	0.041 *
KA	17.94 ± 4.09	21.14 ± 6.49	−1.525	0.131
LA	21.41± 5.46	15.94 ± 6.87	−2.226	0.026 *
**Shortness ()**				
Hamstring (L) **Degree**	19.11 ± 10.49	23.35 ± 12.36	−1.149	0.259
Hamstring (R) **Degree**	21.88± 13.16	26.88 ± 12.35	−1.593	0.111
Pectoral (L) **centimeter**	0.79± 0.91	0.70 ± 1.12	−0.872	0.383
Pectoral (R) **centimeter**	0.82 ± 10.02	0.52 ± 0.94	−1.143	0.253
**Endurance (second)**				
Lateral Bridge (L)	35.54 ± 19.05	38.02 ± 23.096	−0.103	0.918
Lateral Bridge (R)	36.47 ± 20.32	31.08 ± 13.06	−0.396	0.692
Sorensen	38.46 ± 21.68	41.25 ± 27.43	−0.499	0.617
Mc-Gill	45.21 ± 29.54	49.99 ± 6.93	−0.465	0.642

CVA: Craniovertebral Angle; KA: Kyphosis Angle; LA: Lordosis Angle; SD: Standard Deviation; Z: between-group comparison; *p*: Mann–Whitney U Test; *p* * < 0.05.

## Data Availability

The datasets generated and/or analyzed during the current study are not publicly available due to restrictions related to participant confidentiality and institutional data protection regulations. However, de-identified data are available from the corresponding author upon reasonable request.

## Supplementary Materials

The following supporting information can be downloaded at: https://www.mdpi.com/article/10.3390/healthcare14132008/s1, File S1: The CONSORT reporting checklist. References [28,29] are cited in the Supplementary Materials.
